# The dawn of the dual-target era in respiratory disease: promise, pitfalls, and a roadmap for bispecific antibodies

**DOI:** 10.3389/fimmu.2026.1906783

**Published:** 2026-09-14

**Authors:** Yikun Cheng, Jian Ding, Zegeng Li, Jiabing Tong, Cheng Yang

**Affiliations:** 1 First Affiliated Hospital of Anhui University of Chinese Medicine, Hefei, China; 2 Anhui University of Chinese Medicine, Hefei, China; 3 Institute of Respiratory Disease Prevention and Treatment, Anhui Academy of Chinese Medicine, Hefei, China

**Keywords:** asthma, biomarkers, bispecific antibodies, COPD, personalized medicine, type 2 inflammation

## Abstract

Bispecific antibodies targeting two inflammatory pathways are entering respiratory medicine at an unprecedented pace. TSLP/IL-13 bispecifics have progressed into mid-to-late-stage clinical development, with two distinct molecules (lunsekimig and CM512) in Phase II/III and Phase II trials, respectively. Preclinical and early clinical signals indicate that alarmin blockade may extend beyond classical type 2 immunity, offering therapeutic potential in mixed and non-type 2 inflammatory phenotypes. Yet this molecular acceleration risks outpacing our capacity to deploy these agents rationally. No biomarker-guided framework currently exists to determine which patients require dual-pathway versus single-pathway blockade, or which target pair best matches a given patient’s inflammatory endotype. The central clinical tension is whether the incremental benefit of dual-pathway neutralization justifies any increase in immunosuppressive risk. In this Perspective, we argue that the bispecific antibody race may deliver more molecules than necessary unless the field simultaneously builds biomarker-guided matching frameworks. Limited clinical support, drawn mainly from single-target agents such as tezepelumab (efficacy across eosinophil strata) and itepekimab (benefit in former smokers with COPD independent of eosinophils), together with biological rationale, suggests that alarmin blockade may hold promise in mixed and non-type 2 phenotypes; dedicated dual-alarmin trials in these populations remain needed.

## Introduction

The respiratory biologics landscape is undergoing a transformation that few predicted even five years ago. After a decade in which single-pathway monoclonal antibodies (mAbs) against IgE, IL-5, IL-5Rα, and IL-4Rα reshaped the treatment of severe asthma, the field is now advancing rapidly into dual-target territory. AK139, the first IL-4Rα/ST2 bispecific antibody, received IND clearance in early 2025^1^. By early 2026, it had obtained regulatory approval in China to simultaneously launch seven phase II trials across a range of indications^2^, including respiratory diseases such as COPD and severe asthma. Additional bispecific platforms are advancing through development pipelines, including IL-4Rα/IL-33 (ZW1528), which demonstrated potent blockade of IL-4, IL-13, and IL-33 signaling in preclinical models; TSLP/IL-13 (lunsekimig), now in Phase II/III COPD trials (THESEUS and PERSEPHONE); and a fundamentally different “subtractive” CCR3-CD300a approach. More recently, another TSLP/IL-13 bispecific, CM512 has published a first-in-human Phase I study (1) and is currently in Phase II trials for COPD and asthma (2), positioning it as the most advanced agent in this target class after lunsekimig. Furthermore, the TSLP/IL-33 dual-targeting pair is represented by QX031N, which is progressing toward clinical evaluation. To facilitate comparison across these rapidly emerging agents, **Table 1** provides a comprehensive summary of the molecular targets, mechanisms, development stages, advantages, and limitations of dual-target bispecific antibodies and platforms currently in development for respiratory diseases as of mid-2026, including those still in preclinical or IND-enabling stages. Notably, additional additive platforms, such as the IL-4Rα/IL-5 bispecific BBT002 (3) and the TSLP/IL-4Rα bispecific ATI-052^3^, have recently entered Phase Ib evaluation in COPD and asthma, respectively, further expanding the range of target combinations under active clinical investigation.

**Table 1:** Comprehensive Comparative Summary of Dual-Target Bispecific Antibodies in Respiratory Disease (as of mid-2026).

Molecule/platform	Targets	Developer	Development stage	Key respiratory indications	Mechanism type	Advantages	Limitations	Notable data/rationale
AK139	IL-4Rα/ST2	Akeso	Phase II	Severe asthma, COPD	Additive (downstream effector + upstream alarmin)	Simultaneously inhibits IL-4/IL-13 and IL-33; first-in-class; multi-indication Phase II program	No comparative efficacy data; long-term safety unknown	First IL-4Rα/ST2 bispecific to enter clinical trials
Lunsekimig (SAR443765)	TSLP/IL-13	Sanofi	Phase II/III	Eosinophilic COPD (THESEUS, PERSEPHONE); asthma	Additive (alarmin + downstream effector)	Validated Phase Ib PoC in asthma (FeNO reduction); large Phase II/III COPD program	Limited published safety data; efficacy in non-eosinophilic COPD unclear	Positive Phase Ib data; advancing in registrational COPD trials
CM512 (BEL512)	TSLP/IL-13	Keymed Biosciences/Belenos	Phase II	COPD, asthma	Additive (alarmin + downstream effector)	First-in-human Phase I published; most advanced TSLP/IL-13 bispecific after lunsekimig	No published efficacy data; limited safety data	Phase I study; Phase II COPD trial (NCT06980142)
ZW1528	IL-4Rα/IL-33	Zymeworks	Preclinical/IND-enabling	COPD, asthma (planned)	Additive (IL-4/IL-13 + IL-33)	Blocks three cytokines; preclinical suppression of both T2 and non-T2 inflammation; extended half-life	No clinical data; route and dosing not yet established in humans	Preclinical data at ATS/ERS 2025; superior to single-target mAbs in vitro
QX031N	TSLP/IL-33	Qyuns Therapeutics	IND-enabling/Pre-Phase I	Asthma, COPD (planned)	Additive (dual alarmin blockade)	Targets two key epithelial alarmins; potential for broad anti-inflammatory effect	No clinical data; competitive landscape with other alarmin bispecifics	Late-breaking abstract at ERS 2025; advancing toward clinical evaluation
BBT002	IL-4Rα/IL-5	Bambusa Therapeutics	Phase Ib	COPD, asthma, CRSwNP	Additive (IL-4/13 + IL-5 pathways)	Tetravalent format; synergistic blockade of eosinophil development and effector cytokines	Early-phase safety and efficacy data not yet published	Phase I data showed synergistic efficacy in vitro/in vivo; Phase Ib COPD study initiated
ATI-052	TSLP/IL-4Rα	Aclaris Therapeutics	Phase Ib (Proof-of-Concept)	Asthma	Additive (alarmin + IL-4/13 signaling)	Novel pairing of alarmin and downstream effector blockade	Very early development; no efficacy data in patients	Initiated Phase Ib POC trial in asthma
CCR3-CD300a (LC1)	CCR3/CD300a	Academic (Munitz et al.)	Preclinical	Asthma, eosinophilic disorders	Subtractive (inhibitory receptor engagement)	Actively suppresses eosinophils/mast cells; reversed established airway remodeling in mice	No human data; durability and reversibility unknown; off-target inhibitory signaling possible	Complete reversal of chronic asthma pathology in murine models

**Table 1** includes bispecific antibodies that have entered clinical trials (Phase I or later) as of July 31, 2026, plus preclinical candidates with published proof-of-concept data and disclosed development plans. Clinical trial identifiers are provided where publicly available. PoC, proof-of-concept; FeNO, fractional exhaled nitric oxide; T2, type 2; CRSwNP, chronic rhinosinusitis with nasal polyps; IND, investigational new drug; NCT, National Clinical Trial.

This molecular acceleration is, on one level, a triumph of bioengineering. The field has moved from the conceptual demonstration that bispecific antibodies can neutralize two type 2 cytokines simultaneously (pioneered by Godar et al. (4) with their IL-4Rα/IL-5 bispecific in murine house dust mite models) to the design of clinically deployable molecules such as the anti-TSLP/IL-13 “Zweimab” and “Doppelmab” platforms (5), and now to multi-indication Phase II programs. Yet there is a gathering sense that our technological capacity to create these molecules is outstripping our intellectual framework for using them. The core question facing the respiratory community is not whether bispecific antibodies can work—preclinical and emerging clinical data suggest they can—but rather: For which patient, with which endotype, at which stage of disease, does dual-target blockade offer meaningful incremental benefit over refined single-pathway inhibition?

## The strategic divergence: additive blocking versus subtractive modulation

A fundamental strategic divergence is emerging in the bispecific antibody space that deserves careful parsing.

Additive blocking strategies aim to concurrently neutralize two pathogenic cytokine pathways, operating on the premise that redundant and synergistic inflammatory circuits drive persistent disease in patients who respond incompletely to single-pathway blockade. The IL-4Rα/ST2 bispecific AK139 exemplifies this approach, simultaneously inhibiting IL-4/IL-13 signaling (through IL-4Rα) and IL-33 signaling (through ST2). The rationale is biologically compelling: IL-33 is an upstream epithelial alarmin that drives ILC2 activation and type 2 inflammation, while IL-4 and IL-13 are downstream effector cytokines that mediate IgE production, eosinophil trafficking, airway hyperresponsiveness, and mucus hypersecretion. Preclinical work by Ramirez-Carrozzi et al. (6) demonstrated that combined blockade of IL-13 and IL-33 pathways produced greater inhibition of type 2 inflammation than blockade of either pathway alone. Similarly, Toki et al. (7) showed that TSLP and IL-33 reciprocally promote each other’s expression in the lung and upregulate each other’s receptors on ILC2s, creating a positive-feedback loop that may be optimally disrupted by dual targeting.

A comprehensive review of alarmin-targeted bispecific and combination therapies published in late 2025 underscores the biological rationale for targeting two epithelial alarmins positioned upstream of the type 2 cascade, while cautioning that the balance between broader efficacy and safety requires careful prospective evaluation (8). An IL-33/TSLP dual-targeting molecule was presented as a late-breaking abstract at the ERS 2025 Congress (9), further exemplifying this approach. This dual-alarmin strategy draws on the observation that Tezepelumab (the first anti-TSLP mAb approved for severe asthma) reduces exacerbations across a broad spectrum of patients, including those with low eosinophil counts, likely because it intercepts inflammatory signaling at its epithelial origin (10, 11). A hypothesis derived from mechanistic studies is that adding IL-33 blockade to TSLP inhibition might capture the subset of patients in whom IL-33 is the dominant alarmin, but this has not been tested in comparative clinical trials; the biological rationale for such testing is supported by evidence of their reciprocal induction and mutual receptor upregulation on innate lymphoid cells.

A distinct variant of the additive strategy is represented by ZW1528, an IL-4Rα/IL-33 bispecific that blocks three cytokines (IL-4, IL-13, and IL-33) in a single molecule. Preclinical data presented at the American Thoracic Society (ATS) 2025 International Conference and subsequently at ERS 2025 demonstrated that ZW1528 suppressed both type 2 and non-type 2 inflammatory responses in primary human immune cells from COPD patients *in vitro*, and that its blockade of cytokine-driven activation of human epithelial cells was superior to that achieved with mAbs targeting either IL-4Rα or IL-33 alone (12, 13). The molecule incorporates Fc modifications for extended half-life and demonstrates biophysical stability at high concentration (150 mg/mL), supporting subcutaneous administration.

Further expanding the landscape, the TSLP/IL-13 bispecific lunsekimig entered Phase II/III development in 2025 with the THESEUS (NCT07190222) (14) and PERSEPHONE (NCT07190209) (15) trials, which together aim to enroll approximately 1,884 patients with poorly controlled eosinophilic COPD^4^. Lunsekimig previously demonstrated a 40.9 ppb reduction in fractional exhaled nitric oxide versus placebo in a Phase Ib asthma proof-of-concept study, indicating potent suppression of type 2 inflammation (16).

Subtractive modulation represents a fundamentally different philosophy. Rather than merely blocking cytokine ligands or receptors, this strategy uses bispecific antibodies to actively suppress pathogenic effector cells by engaging inhibitory receptors. The CCR3-CD300a bispecific antibody fragment (LC1), developed by Munitz et al. (17), cross-links the activating chemokine receptor CCR3 (highly expressed on eosinophils and mast cells) with the inhibitory receptor CD300a, thereby delivering a dominant-negative signal that actively shuts down effector cell functions. In a murine model of chronic experimental asthma, intranasal LC1 treatment completely reversed established airway inflammation, mucus production, collagen deposition, and peribronchial smooth-muscle thickening (17). The CD300 family of receptors, which contain immunoreceptor tyrosine-based inhibitory motifs (ITIMs), are increasingly recognized as critical regulators of eosinophil survival, chemotaxis, and effector functions (18).

This subtractive approach offers a theoretical advantage: it does not merely starve the inflammatory cascade of cytokine signals but actively suppresses the effector cells that execute tissue damage. It also raises distinct safety considerations—rather than a reversible pharmacologic blockade of a cytokine, it involves an active inhibitory signal delivered to immune cells, the durability and reversibility of which may differ.

## The central clinical tension: is more better, or just riskier?

At the heart of the bispecific antibody dilemma lies an unresolved clinical question: Does dual-pathway blockade meaningfully improve outcomes over optimized single-pathway blockade, and does any such improvement justify the potential for increased immunosuppressive risk?

Consider the benchmark. Tezepelumab, as a single agent targeting TSLP, produced a 71% reduction in annualized exacerbation rates in the overall study population receiving the medium-dose regimen (210 mg every 4 weeks) in the PATHWAY Phase IIb trial, an effect observed regardless of baseline blood eosinophil counts (10) and robust efficacy across eosinophil strata in the Phase III NAVIGATOR trial (11). A 2026 multicenter real-world study further demonstrated that tezepelumab produced sustained multidomain improvements across clinical, functional, and inflammatory domains, with 22.1% of patients achieving strict clinical remission and 73.1% achieving biological remission at 12 months (19). Dupilumab, blocking both IL-4 and IL-13 through IL-4Rα, reduced severe exacerbations by approximately 48% in the overall population and 68% in patients with eosinophil counts ≥300 cells/μL (20). In COPD, pooled data from the BOREAS and NOTUS trials confirmed that dupilumab reduced exacerbations by 28.1% and improved lung function and quality of life in patients with type 2 inflammation (blood eosinophils ≥300 cells/μL), with consistent efficacy even in elderly patients aged ≥65 years (21). These are substantial effect sizes. To demonstrate clear incremental benefit over such established agents, a bispecific antibody must clear a very high bar—and the magnitude of that bar depends on the patient population. For a patient with eosinophilic, type 2-high asthma who achieves near-complete exacerbation suppression with dupilumab or tezepelumab, the added benefit of ST2 or IL-33 blockade may be marginal. Conversely, preclinical evidence and limited early clinical signals suggest that for patients with persistent exacerbations despite single-pathway blockade—or those with mixed inflammatory signatures—dual targeting may offer incremental benefit, although direct head-to-head clinical evidence remains unavailable.

## Safety considerations

Early clinical safety data for respiratory bispecific antibodies, although limited, provide preliminary context. In a Phase Ib proof-of-concept trial of the TSLP/IL-13 bispecific lunsekimig in asthma, the overall incidence of treatment-emergent adverse events was balanced between lunsekimig and placebo groups, with no unexpected safety signals; detailed infection rates and discontinuation data have not yet been fully published. Similarly, developers of AK139 (IL-4Rα/ST2) and BBT002 (IL-4Rα/IL-5) have described Phase I safety profiles as broadly consistent with those of existing single-pathway biologics. Updated preliminary data from the BBT002 multiple-ascending-dose cohorts^5^ presented at EAACI 2026 further support a favorable tolerability profile, while full Phase 1a results for ATI-052 (TSLP/IL-4Rα) reported^6^ in 2026 confirmed good tolerability with no serious adverse events or discontinuations due to treatment-emergent adverse events. Full peer-reviewed long-term datasets remain limited. There are currently no dedicated, publicly available long-term safety databases for any respiratory bispecific antibody. **Supplementary Materials**, **Supplementary Table 1** summarizes the limited available safety information from disclosed trials.

On the risk side, systematic analysis of IL-4/IL-13 pathway inhibition suggests potential concerns including increased risks of certain infections, parasitic reactivation, and possibly cardiovascular events, although the clinical significance of these signals in asthma populations appears modest (22). Broader immunosuppression from simultaneously blocking multiple cytokine pathways, particularly when they include upstream alarmins with pleiotropic functions in tissue repair, anti-helminth immunity, and epithelial homeostasis—raises legitimate questions. IL-33, for instance, plays important roles in tissue repair after lung injury through ST2^+^ Treg-mediated IL-13 secretion (23), and its chronic blockade could theoretically impair mucosal barrier function or anti-viral immunity. Beyond general pathway suppression, bispecific antibodies that simultaneously bind two cell-surface receptors—such as IL-4Rα and ST2—pose distinct pharmacological risks. These include bridging of cells that co-express both targets, potentially triggering unintended cell-cell interactions; avidity-driven selectivity that may alter tissue distribution; and complex target-mediated drug disposition affecting clearance. While well-recognized in T-cell-engaging bispecifics, these phenomena have not been systematically assessed for anti-inflammatory bispecifics in respiratory disease. Reassuringly, these theoretical risks have not manifested as safety signals in the small early-phase datasets available to date, but definitive conclusions require adequately powered Phase III programs with prolonged follow-up.

However, it is important to contextualize these theoretical risks with emerging clinical evidence. A 2025 review by Komori and Ortega (8) examining combined and bispecific biologic approaches in airway disease found that the safety profiles of dual-pathway inhibition appear broadly comparable to those of monotherapies, without unexpected or synergistic toxicities. This suggests that while pharmacovigilance is paramount, dual-target blockade in respiratory disease may not carry the amplified immunosuppressive burden seen with T-cell-engaging bispecific antibodies in oncology, which operate via fundamentally different mechanisms. Nonetheless, as the first respiratory bispecifics enter Phase II/III trials, comprehensive long-term safety data collection from the outset may be essential to confirm this preliminary safety assessment. Should integrated safety analyses from ongoing bispecific programs become available within the next 2–3 years as anticipated, they may provide the first prospective evidence, moving beyond theoretical concerns toward evidence-based risk stratification.

The challenge is that these risks may only become apparent in large, long-term safety databases, the kind that only exist for single-pathway agents with years of post-marketing surveillance. As the first bispecific antibodies enter Phase II and III trials in respiratory indications, the field must prioritize comprehensive safety data collection from the outset.

## The absent framework: toward a biomarker-guided matching architecture

The most striking deficit in the current bispecific antibody landscape is the absence of a biomarker-guided framework for rational patient selection. We have entered an era where we can simultaneously block IL-4Rα and ST2, or TSLP and IL-33, or IL-4Rα and IL-33, or TSLP and IL-13—yet we lack validated biomarkers to determine which of these target pairs a given patient needs.

The existing biomarker armamentarium for respiratory biologics is itself evolving. Blood eosinophil count, fractional exhaled nitric oxide (FeNO), and serum IgE remain useful but imperfect predictors of type 2 inflammation and biologic response. A 2023 pragmatic consensus on biologic selection in severe asthma highlighted the role of these biomarkers while acknowledging their limitations in guiding precise treatment choice (24). They were developed in the context of single-pathway agents and may be inadequate for guiding dual-target therapy. For instance, a patient with a blood eosinophil count of 150 cells/μL but elevated serum IL-33 or ST2 levels might derive outsized benefit from an IL-33-containing bispecific, yet no such biomarker is routinely available. Conversely, a patient with high FeNO and eosinophilia but low TSLP expression might be optimally treated by an IL-4Rα-directed agent alone, without the additional immunosuppressive burden of alarmin blockade.

The concept of treatable traits—pioneered by Agusti et al. (25) —provides a conceptual foundation for the kind of framework needed. This paradigm moves beyond syndromic diagnosis (asthma versus COPD) toward identifying specific, measurable, and modifiable pathological mechanisms in each patient: type 2 inflammation, eosinophilic airway inflammation, mucus hypersecretion, airway hyperresponsiveness, and beyond. A treatable traits approach applied to bispecific antibodies would ask: What are the dominant, measurable pathogenic drivers in this patient’s airway disease, and which target pair optimally neutralizes them? Mappin-Kasirer et al. (26) have articulated how this approach might extend to COPD, where type 2 inflammation is a measurable and modifiable trait in a subset of patients who may benefit from biological therapy. The 2025 approval of mepolizumab as the first anti-IL-5 biologic for eosinophilic COPD—alongside the established role of dupilumab—signals that type 2 biology is clinically meaningful in COPD and reinforces the relevance of biomarker-stratified approaches in this population (27).

The need for biomarker-driven precision is further underscored by a 2025 multi-criteria decision analysis of biologics in COPD, which found that efficacy of pathway-specific biologics remains highly variable across COPD endotypes, and that systematic endotyping is essential to identify patients most likely to benefit from each targeted mechanism (28).

However, moving from concept to clinical implementation requires a substantial investment in: (a) multiparametric biomarker panels that simultaneously measure airway and systemic levels of TSLP, IL-33, IL-4, IL-13, ST2, IgE, eosinophil activity markers, and epithelial integrity markers; (b) dynamic biomarker-response modeling that correlates changes in these biomarkers with clinical outcomes during bispecific therapy; (c) predictive algorithms that identify patients most likely to derive incremental benefit from dual versus single-pathway blockade—an area where multi-omics and machine learning approaches are already demonstrating the feasibility of high-accuracy non-invasive discrimination of asthma inflammatory endotypes (29); and (d) adaptive trial designs that use biomarker stratification to enrich populations for early-phase studies, thereby accelerating the identification of responder profiles.

A potentially instructive example is the experience with dupilumab in COPD, where *post hoc* analyses from BOREAS and NOTUS have demonstrated that efficacy varies according to specific type 2 inflammatory biomarker profiles (30). These data suggest that not all patients with elevated eosinophils are equal, and that more granular biomarker stratification—potentially incorporating FeNO, IgE, and alarmin levels—could further optimize patient selection, an insight directly transferable to the development of bispecific antibodies.

## Translational barriers for alarmin-specific biomarker implementation

The practical implementation of the biomarker-guided framework proposed here faces substantial hurdles. Currently, blood eosinophil count, FeNO, and serum IgE are the only type 2 biomarkers with standardized assays, established reference ranges, and broad clinical availability. In contrast, measurement of circulating or sputum IL-33, ST2, TSLP, and related alarmins remains confined to research laboratories. There are no internationally certified reference materials, no harmonized sample-handling protocols, and substantial inter-assay variability that precludes the definition of validated clinical cut-offs. Furthermore, the cost and throughput of manual ELISA or research-grade multiplex platforms are incompatible with routine clinical laboratory workflows. Without investment in automated, affordable, and standardized alarmin assays—and without prospective trials that validate alarmin-based stratification algorithms—the biomarker-guided deployment of dual-target biologics will remain aspirational and limited to clinical research settings.

Beyond biomarker availability, the widespread adoption of bispecific antibodies will face substantial practical hurdles. The manufacturing complexity of bispecific platforms may initially constrain production capacity, while treatment costs are anticipated to be higher than those of single-pathway biologics, necessitating rigorous cost-effectiveness analyses to justify payer reimbursement. Moreover, regulatory pathways for dual-target agents are still evolving, and ensuring equitable access across different healthcare systems will be a critical challenge that must be addressed in parallel with clinical development.

## A roadmap for rational bispecific deployment

We propose the following roadmap for the rational development and positioning of bispecific antibodies in respiratory disease.

### Phase 1: Endotype deconvolution (now)

The field must invest in comprehensive endotyping of patients who have incomplete responses to existing single-pathway biologics. What inflammatory pathways remain active in dupilumab non-responders? In tezepelumab partial responders? These data will be crucial to define the target product profiles for bispecific antibodies. Pending these data, the first clinical trials of bispecific agents—including the ongoing AK139 Phase II program and the anticipated ZW1528 Phase I program—should include comprehensive biospecimen collection for multi-omics analysis.

### Phase 2: Head-to-head biomarker-defined trials (over the next 2–3 years)

Rather than pitting bispecific antibodies against placebo in unselected populations, trials should use biomarker enrichment strategies. For an IL-4Rα/ST2 bispecific, candidate enrichment biomarkers might include elevated serum ST2 levels, high IL-33 tissue expression, or a transcriptomic signature of IL-33 pathway activation. For an IL-33/TSLP bispecific, enrichment might focus on patients with low eosinophil counts (suggesting non-IL-5-driven inflammation) but high epithelial alarmin activation markers. The ongoing clinical programs for lunsekimig (TSLP/IL-13) in COPD, enriched for eosinophilic phenotype, represent a step in this direction, though more granular endotyping is needed.

### Phase 3: Comparative effectiveness against sequential therapy (over the next 3–5 years)

The relevant comparator for a bispecific antibody may not be placebo but sequential single-pathway therapy: for example, adding an anti-ST2 agent to ongoing dupilumab in incomplete responders. Bispecific antibodies should demonstrate superior efficacy compared with combination therapy, and the convenience of a single injection, while not a primary efficacy driver, represents an additional patient-centered advantage. This principle echoes the evolving approach in severe asthma management, where real-world studies are now evaluating the effectiveness of switching between biologics (31).

### Phase 4: Pharmacovigilance and safety signal detection (ongoing)

Long-term safety registries, modeled on dupilumab post-marketing surveillance, must be established early as these agents advance into Phase III/IV. Prospective surveillance should systematically capture infection rates and any malignancy signals. Importantly, to date, dedicated prospective safety datasets for respiratory bispecifics are lacking; therefore, any claims regarding these risks must be drawn from ongoing Phase II/III programs or analogous biologic registries, rather than extrapolated from theoretical concerns. Effects on mucosal immunity, including potential impacts on anti-helminth defense and epithelial barrier integrity, similarly require active monitoring in longitudinal cohorts. The experience with dupilumab—for which long-term safety data are now emerging from open-label extension studies and real-world registries—provides a practical template for structured post-marketing surveillance.

A critical element across all phases is the integration of biomarker discovery into trial design. The finding that dupilumab’s efficacy in COPD is enhanced in patients with elevated FeNO (≥20 ppb) (32) illustrates the potential for biomarker refinement to identify differential responders—a principle that becomes even more important when multiple pathways are simultaneously targeted. Furthermore, advanced imaging endpoints, which have been proposed as tools to detect structural remodeling changes in biologic-treated COPD (33), should be incorporated into bispecific development programs to accelerate the identification of treatment effects on disease modification. **Figure 1** contrasts the molecular mechanisms of additive cytokine blockade with subtractive effector cell inhibition. **Figure 2** outlines a practical biomarker-driven clinical algorithm to guide the selection of single-pathway monoclonal antibodies versus dual-target bispecific agents according to a patient’s dominant inflammatory endotype.

**Figure 1:**
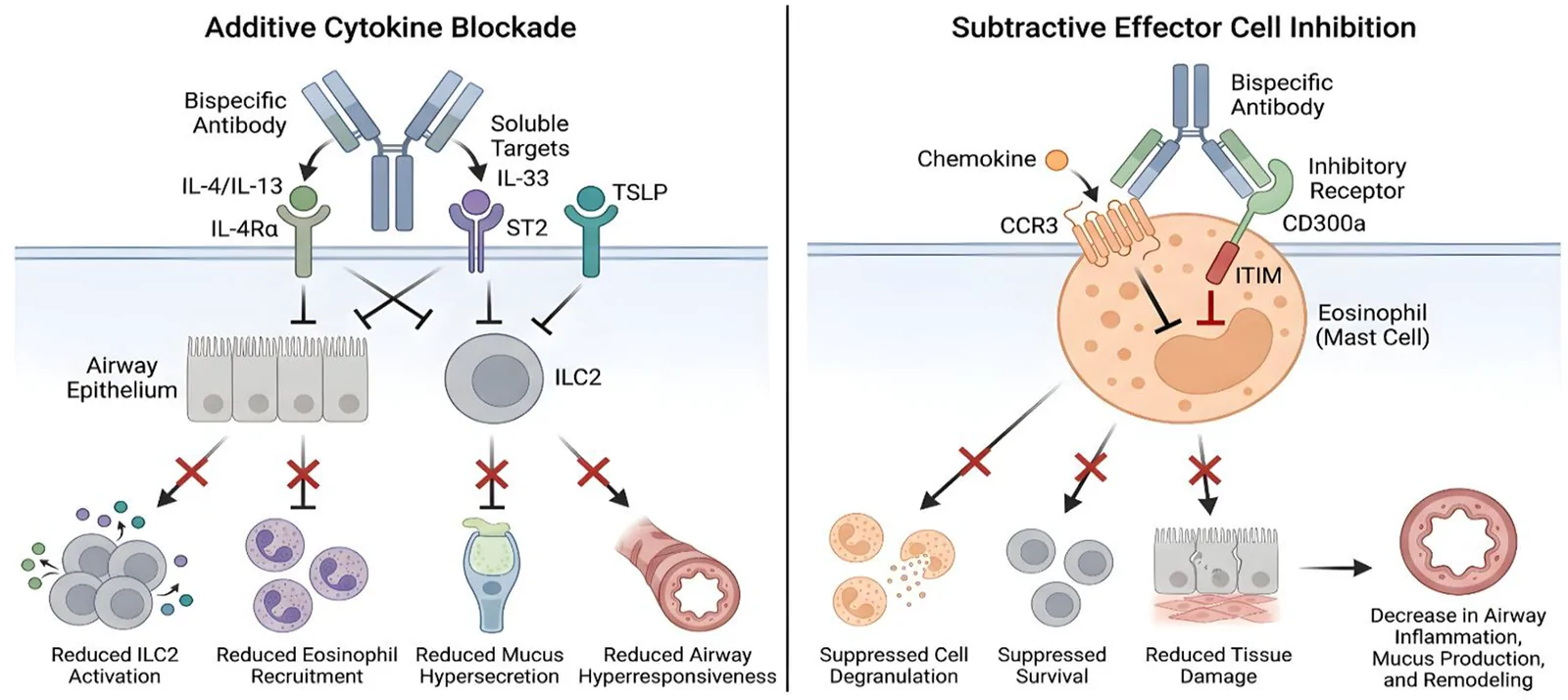
Rational positioning of bispecific antibodies in respiratory disease. Left: Additive cytokine blockade, in which a bispecific antibody simultaneously neutralizes soluble targets (IL-4/IL-13 via IL-4Rα, IL-33 via ST2, and/or TSLP), leading to reduced ILC2 activation, reduced eosinophil recruitment, reduced mucus hypersecretion, reduced airway hyperresponsiveness, and overall decrease in airway inflammation, mucus production, and remodeling. Right: Subtractive effector cell inhibition, in which a bispecific antibody cross-links the activating chemokine receptor CCR3 with the inhibitory receptor CD300a (containing ITIM motifs) on eosinophils and mast cells, resulting in suppressed cell degranulation, suppressed survival, reduced tissue damage, and decrease in airway inflammation, mucus production, and remodeling. IL, interleukin; ILC2, group 2 innate lymphoid cell; ITIM, immunoreceptor tyrosine-based inhibitory motif; ST2, suppression of tumorigenicity 2; TSLP, thymic stromal lymphopoietin.

**Figure 2:**
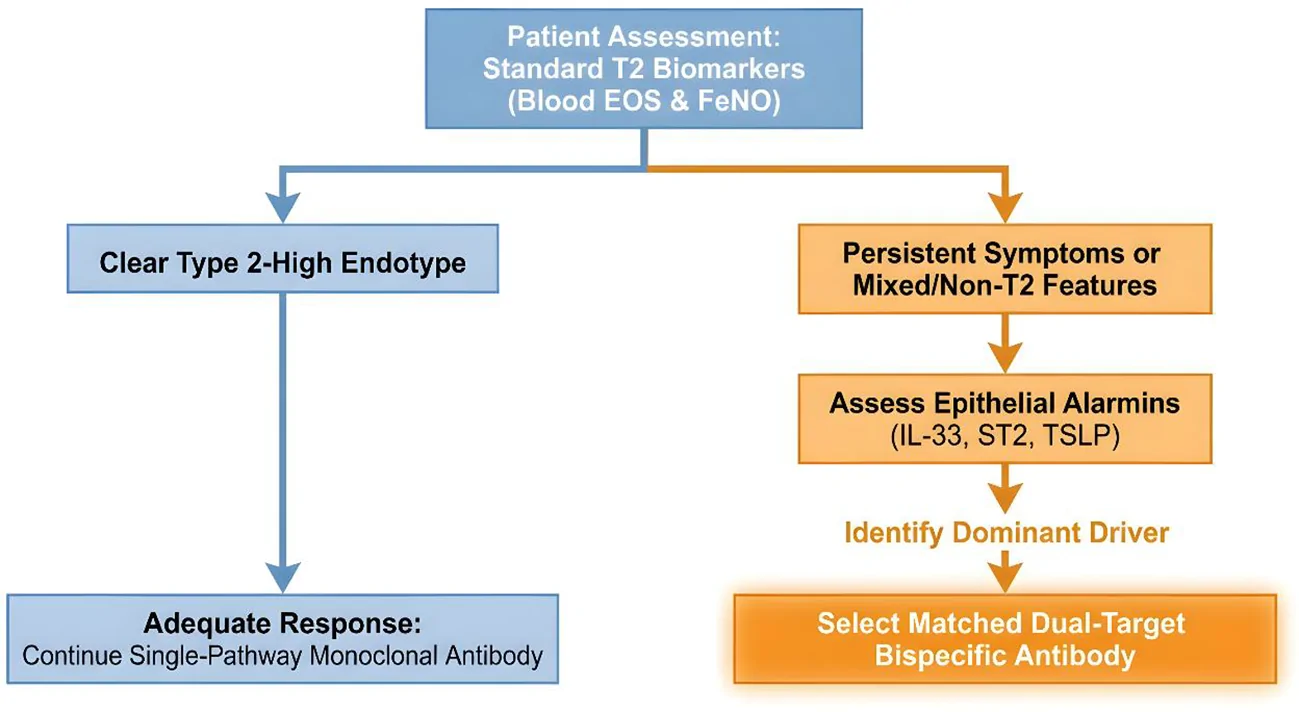
Biomarker-guided clinical algorithm for biologic therapy in airway disease. Patients are first evaluated using standard type 2 (T2) biomarkers (blood eosinophils [EOS] and fractional exhaled nitric oxide [FeNO]). Individuals with a clear type 2-high endotype who achieve an adequate clinical response continue treatment with a single-pathway monoclonal antibody. Patients who experience persistent symptoms or exhibit mixed/non-type 2 features proceed to assessment of epithelial alarmins (IL-33, ST2, and TSLP) to identify the dominant inflammatory driver, followed by selection of a matched dual-target bispecific antibody. This is a conceptual clinical algorithm pending prospective validation and assay standardization for alarmin measurements. EOS, blood eosinophils; FeNO, fractional exhaled nitric oxide; IL, interleukin; ST2, suppression of tumorigenicity 2; T2, type 2; TSLP, thymic stromal lymphopoietin.

## The subtractive advantage: a special case?

The CCR3-CD300a subtractive approach deserves special consideration in this roadmap. Its mechanism—active suppression of effector cells rather than cytokine neutralization—may offer a distinct safety profile and therapeutic advantage. Because it physically eliminates or deactivates the cells that mediate tissue damage, it could theoretically achieve therapeutic effects with less broad immunosuppression than cytokine blockade. Moreover, by targeting a surface receptor combination specific to eosinophils and mast cells, it may achieve cell-type selectivity that cytokine-directed bispecifics cannot.

However, the subtractive approach also faces unique challenges. Its durability and reversibility may differ from conventional biologic therapy, raising questions about dosing frequency and the potential for off-target inhibitory signaling. And while the preclinical data from Munitz et al. (17) are impressive—complete reversal of established airway remodeling—this finding has not yet been independently replicated in human trials. The respiratory community should watch this space closely, as a successful subtractive bispecific could fundamentally change how we think about treating airway inflammation.

## Beyond asthma: the COPD frontier

The extension of bispecific antibodies into COPD represents one of the most exciting—and uncertain—frontiers. The initiation of AK139 for COPD Phase II trials, the initiation of the THESEUS and PERSEPHONE trials for lunsekimig in eosinophilic COPD, and the progression of ZW1528 toward clinical testing all signal a conviction that dual-target blockade may be particularly valuable in this heterogeneous disease, where type 2 inflammation is present in only a subset of patients but may coexist with neutrophilic, type 1, or type 3 inflammatory patterns. In patients exhibiting features of both asthma and COPD—commonly referred to as asthma-COPD overlap (ACO)—managing mixed inflammatory profiles is especially challenging due to variable endotypes and structural remodeling. In COPD, the argument for bispecific antibodies may be even stronger than in asthma: by simultaneously blocking a broader range of inflammatory pathways, dual-target agents might capture a larger proportion of patients with mixed inflammatory endotypes. A recent review (34) underscores this point, noting that while current biologics (anti-IgE, anti-IL-5, anti-IL-4Rα/IL-13, and anti-TSLP) show efficacy in T2-high asthma and biomarker-selected COPD, evidence in ACO remains limited and derived mainly from separate disease studies; no agent is yet approved specifically for ACO. Emerging dual alarmin blockers and IL-33/ST2 targeting hold particular promise for mixed phenotypes. This is supported by the efficacy of the anti-IL-33 antibody itepekimab in former smokers with COPD, which appeared independent of baseline eosinophil levels, indicating benefit beyond classical type 2 inflammation. Clinical support remains limited to indirect evidence from single-target alarmin blockade (e.g., tezepelumab across eosinophil strata and itepekimab in former smokers with COPD independent of eosinophils); dedicated dual-alarmin trials in non-type 2 or mixed phenotypes are still needed. Bispecific antibodies that simultaneously neutralize two pathogenic nodes may therefore be ideally positioned to address the overlapping inflammatory drivers characteristic of ACO, offering a single-agent solution for a dual-disease challenge.

A 2025 review of the emerging role of alarmin-targeting biologics in COPD articulates this rationale: given the heterogeneity of COPD pathophysiology, it is likely that certain biologics will benefit only specific COPD endotypes, and ongoing trials must provide information about which groups benefit from blockade of which specific pathways (33). ZW1528’s preclinical demonstration of suppressing both type 2 and non-type 2 responses in primary human immune cells from COPD patients is particularly intriguing in this context, as it suggests that dual IL-4Rα/IL-33 blockade may address the mixed inflammatory signatures frequently encountered in this disease (12, 35).

Yet the COPD experience also underscores the biomarker imperative. The failure of multiple biologic agents in unselected COPD populations highlights the risk of testing targeted therapies without enrichment strategies. The success of dupilumab in eosinophilic COPD, with pooled BOREAS and NOTUS data showing a number needed to treat of approximately 2–3 to prevent one exacerbation (36), provides a proof of concept that biomarker-guided biologic therapy works in COPD, but it also raises the bar for bispecific antibodies, which must demonstrate incremental value over this new standard of care.

Moreover, the recent FDA approval of depemokimab (December 2025)^7^, the first ultra-long-acting biologic dosed twice yearly, which demonstrated 58% and 48% reductions in annualized exacerbation rates in the SWIFT-1 and SWIFT-2 trials respectively (37), further illustrates that the single-pathway biologics landscape continues to evolve rapidly, with improvements in dosing convenience that may partially offset the “one injection versus two” argument for bispecifics. Bispecific developers must account for these moving benchmarks when designing comparative trials.

## Disease-specific nuances of a biomarker-guided strategy

Although a unified treatable-traits framework conceptually spans asthma, COPD, and asthma-COPD overlap (ACO), the pathophysiological drivers differ substantially, requiring disease-specific adaptations. In asthma, type 2 inflammation is dominant and well captured by EOS and FeNO, making them relatively reliable guides for biologic selection. In COPD, eosinophilia is only one component of a heterogeneous inflammatory landscape that frequently includes neutrophilic, type 1, and type 3 signatures, and the same eosinophil threshold does not carry identical predictive weight as in asthma. ACO adds further complexity by combining variable airflow obstruction with structural remodeling, likely necessitating a composite biomarker approach. No bispecific antibody trial has yet enrolled a dedicated ACO cohort with endotype-guided stratification. Therefore, the biomarker algorithm proposed in **Figure 2** must be considered a starting template; disease-specific validation cohorts are an urgent priority.

## Conclusions

The bispecific antibody era in respiratory medicine is both inevitable and welcome. Notably, innovations in antibody engineering and pulmonary delivery systems, such as inhaled bispecific formulations (38), may further mitigate systemic risks and enhance real-world applicability across asthma, COPD, and ACO phenotypes. By confining drug exposure primarily to the airways, inhaled bispecifics could directly reduce the systemic immunosuppressive burden that Phase 4 pharmacovigilance programs are designed to detect, providing a complementary safety strategy to biomarker-guided patient selection. The molecular technologies now available are extraordinary, enabling the creation of molecules that simultaneously engage two pathogenic pathways with high affinity and specificity. The biological rationale for dual targeting is supported by a substantial body of preclinical evidence demonstrating pathway synergy and redundancy, together with an emerging armamentarium of clinical-stage molecules that show promising preclinical profiles and early clinical signals. And the clinical need is clear: despite the success of single-pathway biologics—with tezepelumab achieving multidomain clinical remission in approximately one-fifth to one-third of severe asthma patients and dupilumab demonstrating meaningful but incomplete exacerbation reductions in COPD—many patients remain symptomatic or exacerbation-prone.

Several critical gaps must be addressed before routine implementation is feasible: (1) direct comparative effectiveness of dual- versus single-pathway blockade remains unproven; (2) long-term safety databases must be established prospectively; (3) validated, clinically accessible biomarker algorithms are lacking; (4) the optimal positioning of bispecifics in treatment sequences requires head-to-head trials; and (5) disease-specific efficacy in ACO and non-type 2 COPD awaits dedicated endotype-guided studies. Priority should be given to biomarker-stratified adaptive trials and the creation of multi-center safety registries. The respiratory community—clinicians, scientists, regulators, and industry partners—must invest in the endotyping infrastructure, the multiplex biomarker platforms, the machine-learning-enabled predictive algorithms, and the adaptive trial designs that will allow us to match the right bispecific antibody to the right patient at the right time.

## Data Availability

The original contributions presented in the study are included in the article/**Supplementary Material**. Further inquiries can be directed to the corresponding authors.

## Footnotes

1
Akeso Announces IND Acceptance For AK139, A Bispecific Antibody Targeting IL-4Rα And ST2 For Respiratory And Skin Diseases

2
Akeso's IL-4Rα/ST2 Bispecific Antibody Cleared for Seven Phase II Studies in China Spanning Respiratory and Autoimmune Indications

3 Aclaris corporate presentation. Aclaris (2025). Available online at: https://investor.aclaristx.com/static-files/e0078ebd-0054-4ef2-ab2e-a8bdeb72356d (Accessed July 24, 2025).

4 Sanofi Initiates Phase II/III Trials for TSLP/IL-13 BsAb lunsekimig in COPD (THESEUS and PERSEPHONE). PharmCube Global Drug News. September 27, 2025. https://global.pharmcube.com/news/detail/e456e40dce294a779b4459b9d79ab384?type=dailyNews

5
Bambusa Therapeutics Presents Positive Preliminary Multiple Ascending Dose Phase 1 Data for BBT002 at the European Academy of Allergy & Clinical Immunology (EAACI) Annual Congress 2026 - Bambusa Therapeutics

6
sec.gov/Archives/edgar/data/1557746/000110465926049733/acrs-20260428xex99d2.htm

7
FDA Approved Depemokimab to Treat Severe Asthma Characterized by An Eosinophilic Phenotype as an Add-on Maintenance Therapy - Immune System Research
