# Supplementary material for: The dawn of the dual-target era in respiratory disease: promise, pitfalls, and a roadmap for bispecific antibodies
Source: Front Immunol. 2026 Sep 14;17:1906783. doi: 10.3389/fimmu.2026.1906783 (PMC13616614; doi:10.3389/fimmu.2026.1906783)
Supplement: Supplementary file 1 [file DataSheet1.doc]

Table S1. Summary of Currently Available Clinical Safety Data for Respiratory Bispecific Antibodies (as of mid-2026)

| **Molecule** | **Targets** | **Development Phase (as of mid-2026)** | **Trial Population** | **Key Safety Findings** | **Infection Rates** | **Discontinuation Due to AEs** | **Reference/Source** |
| --- | --- | --- | --- | --- | --- | --- | --- |
| Lunsekimig (SAR443765) | TSLP / IL‑13 | Phase Ib (completed); Phase II/III ongoing | Adults with asthma (Phase Ib PoC) | Overall TEAE incidence balanced between lunsekimig and placebo; no unexpected safety signals identified | Not yet disclosed separately from overall TEAE data | Not yet disclosed | Deiteren et al., Eur Respir J 2025;65(4) |
| AK139 | IL‑4Rα / ST2 | Phase I completed; Phase II ongoing | Healthy volunteers / patients (exact numbers not publicly disclosed) | Phase I safety profile described as broadly consistent with existing single‑pathway biologics; no novel safety concerns reported | Not disclosed; reported as “consistent with approved biologics” | Not disclosed | Developer press releases; conference abstracts (no peer‑reviewed publication yet) |
| BBT002 | IL-4Rα / IL-5 | Phase I completed; Phase Ib ongoing | Healthy volunteers; COPD (Phase Ib) | Phase 1 SAD/MAD data (EAACI 2026) showed good tolerability and mostly mild-to-moderate TEAEs; full peer-reviewed safety data not yet published. | Not disclosed separately | Not disclosed | Ho et al., Am J Respir Crit Care Med 2025;211:A1302 (molecular characterisation); Bambusa Therapeutics EAACI 2026 presentation (preliminary MAD safety) |
| ATI-052 | TSLP / IL-4Rα | Phase 1a completed; Phase Ib (ongoing) | Healthy volunteers (Phase 1a); Asthma (Phase Ib PoC) | Phase 1a SAD/MAD (2026) demonstrated favourable safety, mainly Grade 1 injection‑site reactions, with no serious AEs or conjunctivitis events. | Not yet disclosed in detail | None reported in Phase 1a | Aclaris Therapeutics Phase 1a topline results (2026); corporate presentations and press releases |
| ZW1528 | IL‑4Rα / IL‑33 | Preclinical / IND‑enabling | Not yet in human trials | No clinical safety data available | N/A | N/A | Preclinical studies only; Poffenberger et al., Am J Respir Crit Care Med 2025;211:A3446 |
| QX031N | TSLP / IL‑33 | Preclinical / IND‑enabling | Not yet in human trials | No clinical safety data available | N/A | N/A | Developer pipeline disclosures; no clinical data |
| CCR3‑CD300a (LC1) | CCR3 × CD300a (subtractive) | Preclinical | Murine models only | No human safety data; theoretical concerns relate to durability of inhibitory signaling | N/A | N/A | Munitz et al., J Allergy Clin Immunol 2006;118:1082‑1089 |

Abbreviations: TEAE, treatment‑emergent adverse event; AE, adverse event; PoC, proof‑of‑concept; IND, investigational new drug; N/A, not applicable.

Note: All safety data are preliminary and derived from early-phase trials, conference presentations, or press releases. No dedicated long-term safety database exists for any respiratory bispecific antibody as of July 31, 2026. Updated safety data from 2026 presentations (including BBT002 MAD data at EAACI 2026 and full ATI-052 Phase 1a results) are available and currently consistent with the safety profiles of approved single-pathway biologics; however, definitive conclusions require adequately powered Phase III/IV programs with extended follow-up. A recent review noted that preliminary dual-pathway safety profiles appear broadly comparable to monotherapies [8].
